# Co-Expression of T- and B-Cell Markers in a Canine Intestinal Lymphoma: A Case Report

**DOI:** 10.3390/ani12243531

**Published:** 2022-12-14

**Authors:** Pâmela Cristina Lopes Gurgel Valente, Maria Conceição Peleteiro, Sandra Carvalho, Rodolfo Oliveira Leal, Constança Pomba, António Duarte, Jorge Correia

**Affiliations:** 1 Centre for Interdisciplinary Research in Animal Health (CIISA), Faculty of Veterinary Medicine, University of Lisbon, 1300-477 Lisbon, Portugal; 2 Associate Laboratory for Animal and Veterinary Sciences (AL4AnimalS), 1300-477 Lisbon, Portugal

**Keywords:** dog, intestinal lymphoma, enteropathy, co-expression, CD3, CD20, clonality

## Abstract

**Simple Summary:**

The diagnosis of chronic gastroenteropathy in dogs can be complex, highlighting the relevance of histopathology allied to immunohistochemistry in the identification of the involved cell types, contributing to the exclusion of differential diagnoses, including special types of intestinal lymphomas. However, rare cases of the co-expression of some immunomarkers in lymphoid cells can make the interpretation of results particularly difficult. This report presents a case of a dog with a diagnosis of intestinal lymphoma, in which the co-expression of CD3 and CD20 in lymphoid cells was identified in immunocytochemistry and immunohistochemistry, making it necessary identify lymphoid clonality using polymerase chain reaction for antigen receptor rearrangement (PARR) for a precise diagnosis.

**Abstract:**

An 8-year-old female neutered Labrador retriever was presented for a second opinion consultation due to vomiting and lethargy, having failed to respond to symptomatic therapy. Blood analysis revealed hyperbilirubinemia and hypoalbuminemia, associated with hypocobalaminemia. An abdominal ultrasound identified diffused bowel thickening and hypoechoic hepatomegaly. An ultrasound-guided liver fine-needle aspiration was performed for cytology and also for cell block immunocytochemistry. Gastric and duodenal biopsies were collected by gastroduodenoscopy. Liver cytology showed numerous lymphocytes, suggesting lymphoma at the hepatic infiltration stage, and immunocytochemistry in the cell block of the hepatic aspirate indicated co-expression of CD3 and CD20 in the lymphoid cells present. The histopathology of gastric and duodenal biopsies supported the hypothesis of gastrointestinal lymphoma due to heavy lymphoid infiltration of the gastric epithelium and intestinal mucosa, including the villi. Concurrent immunohistochemistry was performed using CD3, CD20, PAX5, and CD79αcy antibodies. Immunomarking was positive for CD3 and CD20, which overlapped populations of lymphoid cells, and was negative for all other antibodies. In the clonality test, lymphocyte co-expression of CD3 and CD20 was confirmed by monoclonal rearrangement of T-cell gamma receptors. The final diagnosis was type 2 enteropathy-associated T-cell lymphoma with hepatic infiltration. Co-expression was examined in conjunction with the PARR result in the presence of T-cell monoclonal rearrangement.

## 1. Introduction

Primary gastrointestinal tract lymphoma, although less frequent in dogs than in cats, is cited as one of the main neoplastic causes of chronic gastroenteropathy in domestic animals [1,2]. It is most commonly reported in the small intestine, stomach, and colon. Several segments of the intestine may be involved, and there is often a dispersal to regional lymph nodes and the liver [3,4,5].

Currently, it is known that this neoplasm originates mainly from T-lymphocytes in dogs and cats [1,2,3]. When lesions are not transmural or histopathology is based on endoscopic biopsies, the diagnosis is considered a challenge, requiring more than the conventional histopathological evaluation to differentiate it from inflammatory bowel disease (IBD) [2].

In humans, the histopathological classification of the World Health Organization subdivides enteropathies associated with intestinal T-cell lymphoma (EATL) into types 1 and 2. This classification is also used in cats and, more recently, in dogs [6]. Type 1 EALT is composed of large cells associated with necrosis and an inflammatory background, whereas type 2 is monomorphic median to small-sized cells with no inflammatory background and rarely with necrosis [2,6,7,8]. Based on this classification, the most common primary intestinal lymphoma in cats is T-cell-associated type 2 enteropathy with marked epitheliotropism, which may be morphologically indistinguishable from IBD. In dogs, a higher frequency of cases associated with type 1 enteropathy is described, with or without accompanying inflammation and/or epitheliotropism, which makes these parameters much less important in the diagnosis of this species [2,4,6].

With the combination of histopathological and immunohistochemical evaluations, the ability to differentiate intestinal lymphomas from IBD has improved. With these methods, it is expected that most neoplastic cells from intestinal lymphoid tumors will express either one of the T- or B-cell markers, but not both. However, there are already some reports of co-expression of CD3 and CD20 in canine lymphomas, including three cases of intestinal T-cell lymphomas associated with type 1 EATL [9,10,11]. These findings reinforce the importance of PCR for antigen receptor rearrangement (PARR) for a definitive diagnosis of lymphoid cells in intestinal neoplasms [1,2,6,10].

The objective of this work is to present the case of a dog with intestinal lymphoma with co-expression of CD3 and CD20, compatible with EATL type 2, that was also showing hepatic infiltration.

## 2. Case Presentation

A neutered 8-year-old female Labrador retriever was presented to the Veterinary Teaching Hospital (HEV), Faculty of Veterinary Medicine, University of Lisbon for a second opinion consultation due to lethargy, anorexia, and vomiting for 10 days, refractory to symptomatic treatment. Blood tests had been previously performed by the referring veterinarian, showing a normal complete blood count, increased alanine aminotransferase (ALT) 185 IU/L (RI: 10–78 IU/L), alkaline phosphatase (ALP) 188 UI/L (RI: 7–83 UI/L), total protein 4.9 g/dL (RI: 5.4–7.8 g/dL), and hypoalbuminemia albumin 1.7 g/dL (RI: 2.2–3.5 g/dL). The animal was hospitalized for symptomatic treatment with intravenous fluids and symptomatic treatment including metronidazole (10 mg/Kg every 12 h), amoxicillin with clavulanate (20 mg/Kg every 12 h), omeprazole (1 mg/Kg every 12 h), maropitant (1 mg/Kg every 24 h), and dexametasone (0.2 mg/Kg every 24 h).

In the physical examination, the dog was prostrated, with pink mucous membranes, normal abdominal palpation, no peripheral lymph node enlargement, and the presence of hematochezia. The capillary refill time (CRT) was 2 s, the heart rate was 168 bpm, the respiratory rate was 16 cpm, the pulse was strong, and the temperature was 39.2 °C. At this point, the differential diagnosis for acute vomiting was established, detailing a digestive cause (infectious gastroenteritis, protein-losing enteropathy, primary vs. secondary lymphangiectasia caused by inflammatory bowel disease or neoplasia). An extra-digestive origin, such as liver disease, pancreatitis, or protein-losing nephropathy, was not excluded at this stage. Taking into account increased liver enzymes, primary (toxic, infectious, or copper-associated hepatopathy or neoplasia) or secondary (reactional hepatopathy secondary to gastroenteropathy, for instance) hepatopathies were considered.

The animal was admitted for medical investigation, and blood and urine samples were collected for a new biochemistry assessment and urine analysis. Results showed hypoalbuminemia (1.49 g/dL, RI: 2.2/3.5 g/dL), hyperbilirubinemia (1.17 mg/dL, RI: 0.0/0.41 mg/dL), hypocobalaminemia (172 ng/L, RI: 275–590 ng/L), and increased canine-specific pancreatic lipase (cPLI), 418 μg/L (RI: 201–399 μg/L). Apart from bilirubinuria, urine analysis was unremarkable, and the UPC was negative (0.18).

After evaluating the new results, a search for vector-borne diseases was also carried out (leishmaniosis, dirofilariosis, borreliosis, anaplasmosis, and ehrlichiosis) and found to be consistently negative.

An abdominal ultrasound exam was performed, which identified hypoechoic hepatomegaly with mild thickening of the gallbladder wall, stomach distention with marked hypomotility, and diffuse thickening of the entire intestine associated with small striations in the duodenum and jejunum, with maintenance of layering. Hypoechogenic mesenteric lymph nodes were at the upper limit in terms of size, and the kidneys showed small calcifications. Mucosal striations associated with hypoproteinemia and hypoalbuminemia supported a possible protein-losing disease. At this point, taking into account the concurrent context of hypoalbuminemia, the main differentials were a protein-losing enteropathy secondary to intestinal inflammation versus infiltrative neoplastic disease (involving the gastrointestinal and hepatobiliary tracts).

Given the suspicion of liver disease associated with an exudative enteropathy (primary, secondary, inflammatory, or neoplastic disease), an ultrasound-guided liver FNA was performed for a cytological examination and for cell block preparation with Histogel^TM^ for immunocytochemistry.

The liver cytological analysis showed numerous plaques of generally normal-sized hepatocytes, among which numerous lymphoid cells were observed, corresponding to a homogeneous population of small-to-medium-sized cells with a slightly indented nucleus of homogenous chromatin and no clearly evident nucleoli. Neutrophils were also observed, albeit in reduced numbers, together with rare macrophages with phagocytosed material in their cytoplasm. The cytologic diagnosis suggested an infiltrative lymphocytic proliferation.

Due to the presence of hypoalbuminemia, hypocobalaminemia, and suspected protein-losing enteropathy, a gastroduodenoscopy and colonoscopy (for ileal sampling) were suggested, but, due to the non-neglectable prolonged anesthetic risk, only the gastroduodenoscopy was performed. This exam revealed signs of erosive gastritis. The duodenal mucosa was irregular and particularly friable, showing diffusely increased granularity. Multiple duodenal and gastric biopsies were performed.

**Figure 1:**
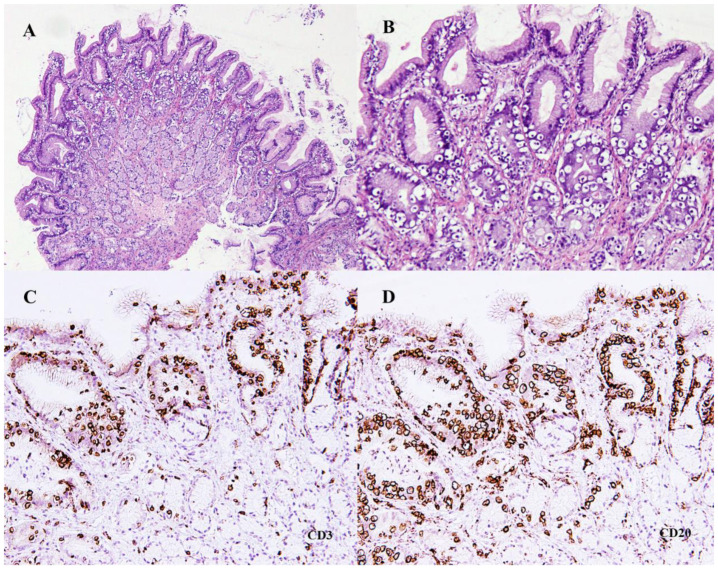
(**A**,**B**) Microphotographs of endoscopic biopsies of gastric pyloric mucosa showing infiltration of the superficial epithelium and one of the crypts by small lymphocytes (H&E, (**A**) ×40 and (**B**) ×100). (**C**,**D**) Immunohistochemistry for lymphoid T-cells (**C**) (anti-CD3) and B-cells (**D**) (anti-CD20) (Mayer’s hematoxylin, ×100).

**Figure 2:**
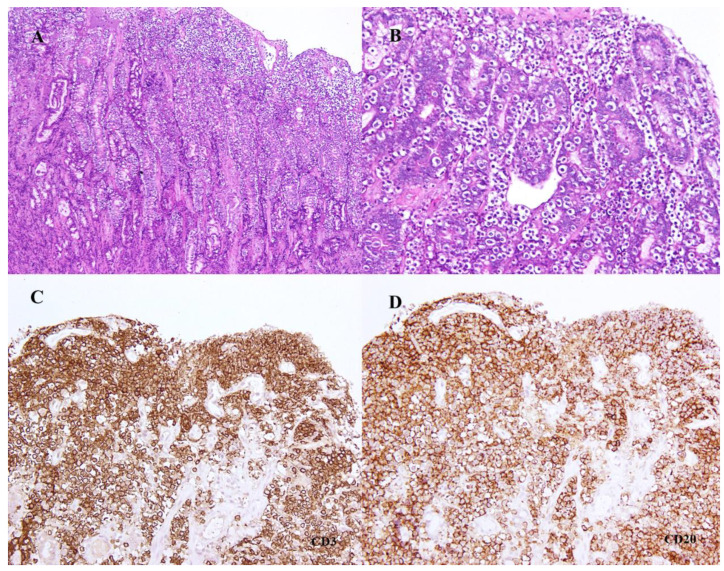
(**A**,**B**) Microphotographs of endoscopic biopsies of duodenal mucosa showing severe infiltration of the epithelium of the villi by small lymphoid cells identical to the ones in the gastric mucosa (H&E, (**A**) ×40 and (**B**) ×100). (**C**,**D**) Immunohistochemistry for lymphoid T-cells (**C**) (anti-CD3) and B-cells (**D**) (anti-CD20) (Mayer’s hematoxylin, ×40).

The histopathological analysis of the biopsies revealed, in the pyloric area of the stomach, marked surface irregularities with anfractuous crypts. The lamina propria showed moderate fibrosis with a decrease in the number of glands and mild inflammatory infiltration, mostly by mononuclear cells. Small, round lymphoid cells with clear cytoplasm were seen intensely infiltrating the lining epithelium and that of the crypts (Figure 1A,B). No microbial agents were identified. In the duodenum, the mucosa showed marked infiltration of the lamina propria by the same type of small lymphoid cells with clear cytoplasm, more intense in the villi, which were shortened and fused. These small lymphoid cells were also heavily present in the villi epithelium (Figure 2A,B), similarly to what was seen in the pyloric epithelium.

The histopathological diagnosis was chronic fibrous gastritis with hyperplasia of the epithelium in the pyloric antrum and severe epithelial infiltration by small lymphoid cells as well as neoplastic infiltration of the duodenum by the same type of small lymphoid cells with marked epithelial tropism in the villi, consistent with intestinal lymphoma.

Immunophenotyping of lymphoid cells in the biopsies and in the hepatic cell block prepared from the liver aspirate was performed using the EnVisionTM Kit (Dako, Agilent, Santa Clara, CA, USA) protocol with the following antibodies: anti-CD3 (polyclonal antibody, Dako, dilution 1:400) as a T-cell marker and anti-CD20 (polyclonal antibody, Biocare Medical, Pacheco, CA, USA, 1:50), PAX5 (monoclonal antibody, SP34 clone, Ventana, Tucson, AZ, USA, ready-to-use), and anti-CD79αcy (monoclonal antibody, HM57 clone, Dako, 1:200) as B-cell markers.

Immunohistochemistry was accomplished at a PTLink station (Dako). Antigen retrieval was achieved with concentrated Tris/EDTA pH 9.0 for all antibodies except PAX5, for which retrieval was achieved with citrate buffer pH 6.0. Mayer’s hematoxylin was used for background staining.

Immunohistochemistry showed strong CD3-positive cytoplasmic and membrane marking in 80% of the small lymphoid cells in both stomach and duodenal mucosae (Figure 1C and Figure 2C). Additionally, there was also strong cytoplasmic and membrane marking for CD20 (Figure 1D and Figure 2D) in a slightly smaller percentage of the lymphoid cells, estimated at 70%, with a clear overlap with the CD3 marking. There was no immunoreactive labelling for PAX5 and CD79αcy. The cell block prepared from the hepatic aspirate also revealed the co-expression of CD3 and CD20 in the lymphoid cells present.

**Figure 3:**
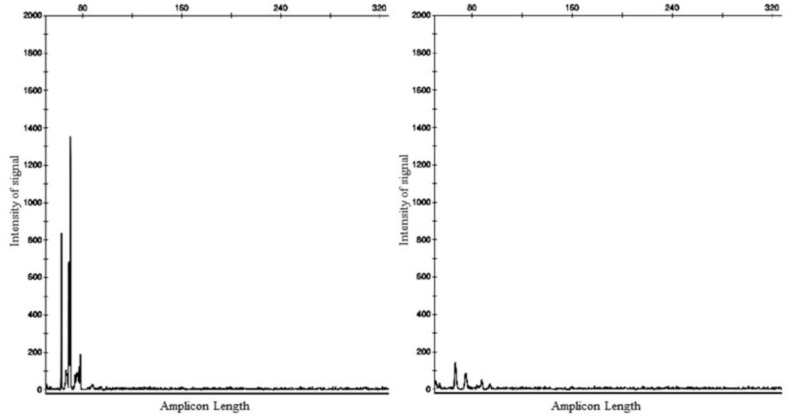
Molecular clonality analysis performed on DNA extracted from paraffin blocks using capillary electrophoresis in the 3500 Genetic Analyzer (Applied Biosystems^®^). Results show clonal amplification with a 55–82 bp peak of the T-cell receptor (TCRγ) on the lower left and negative or poor amplification for the B-cell receptor (IgH) on the lower right after using the PARR technique protocol. The x-axis is the length of the amplicon, and the y-axis is the intensity of the signal.

Subsequently, the polymerase chain reaction for antigen receptor rearrangement (PARR) technique was performed using DNA extracted from paraffin blocks of the stomach and duodenum for B- and T-cells. The analysis revealed the presence of monoclonal rearrangement of the T-cell gamma receptor gene and did not amplify for B-cell receptors [12] (Figure 3).

A definitive diagnosis of intestinal T-cell lymphoma with concomitant expression of B-cell markers and hepatic infiltration was finally issued. The prognosis was considered reserved.

Due to the intense weakness and the unfavorable prognosis, euthanasia of the dog was requested by the owners and necropsy was not authorized.

## 3. Discussion

This case report is a good example of the complexity of the diagnosis of chronic gastroenteropathy in dogs, highlighting the importance of employing the use of histopathology with immunohistochemistry and clonality assays in the identification of the cell types involved, contributing to a definitive diagnosis.

In the present case, the clinical signs, the analytic abnormalities, the poor response to therapy, and the ultrasound changes in the stomach, intestine, and liver were highly suggestive of severe enteropathy associated with reactive and/or neoplastic liver disease.

Hypoalbuminemia and hypoproteinemia can occur in cases of intestinal lymphomas in dogs [4,13,14]. The presence of hyperbilirubinemia and bilirubinuria without anemia, associated with a concurrent increase in liver enzymes, supported a hepatic cause, in this case, a consequence of the infiltrative neoplastic disease in the liver parenchyma [15].

In view of the suspicion of liver disease associated with enteropathy, and recognizing the potential limitations of this exam, a liver FNA was prioritized due to the poor health condition of the animal. The diagnosis of liver cytology was essential to address the possibility of gastrointestinal lymphoma, mainly because this organ is frequently affected in this neoplasm, and cytology may be useful and quite sensitive for the diagnosis of round-cell hepatic tumors [3,4,5]. On the basis of liver cytology, the diagnosis of lymphoma is usually straightforward when it involves large cells. However, the presence of a small-to-intermediate-sized lymphocyte population made it necessary to carry out additional diagnostic tests, mainly to rule out possible differential diagnoses such as lymphocytic hepatitis [5,16].

Concerning the GI tract, the hypothesis of intestinal lymphoma was confirmed by histological analysis of the endoscopic biopsies of the stomach and intestine. In fact, the heavy lymphoid infiltration in the intestine was consistent with what is generally described for intestinal lymphoma, although a differential diagnosis with lymphoplasmocytic enteritis is always considered [2].

It is generally accepted that histopathological evaluation of incisional biopsies has greater value compared to endoscopic biopsies in the diagnosis of enteropathies, considering that all layers of the gastrointestinal wall can be evaluated [4], facilitating the differential diagnosis of IBD versus enteropathy-associated T-cell lymphoma type 2 [6]. However, the use of endoscopic biopsies is commonly accepted in clinical practice as less invasive and is especially recommended in debilitated animals. In the present case, the histopathological examination of endoscopic gastric and intestinal biopsies was a step further in the confirmation of the diagnosis of gastrointestinal lymphoma, with some uncommon findings, such as the marked presence of lymphoid cells in the gastric epithelium without infiltration of the mucosal *lamina propria*. In fact, the severe infiltration of the intestinal mucosa with a monomorphic lymphocyte population was also present in large numbers in the villous epithelium, obliterating the *lamina propria*:epithelial boundary, clearly suggesting lymphoma [5]. Considering the small size of the lymphoid cells and the absence of necrosis and an inflammatory background, the diagnosis of EALT type 2 was appropriate.

Immunohistochemistry is usually quite helpful in the differential diagnosis of IBD and intestinal lymphoma, as in IBD, positive labelling should be mixed between T- and B-cells [6]. Therefore, it was surprising to find the labelling of the lymphoid cells with both T- and B-cell markers, with the same occurring in the cell block that was prepared from the cells obtained in the liver puncture. Cell blocks, prepared from aspirates of organs and tissues, are considered an excellent minimally invasive technique for the characterization and immunophenotyping of canine lymphomas [17,18]. They are also an excellent alternative to cytology immunostaining when incisional biopsies are not possible as the comparison between antibody labelling can be accurate: the exact same cells are being evaluated regardless of the number of antibodies used [19].

Co-expression of CD3 and CD20 confirmed by immunohistochemistry is rare and has been described in only a few cases of canine T-cell lymphomas, such as cutaneous, intestinal, nodal, and peripheral lymphomas with infiltration of the heart and peripheral nerves [9,10,11,20]. Double lymphoid labelling has also been described in a cat, but in a B-cell lymphoma with expression of CD3, identified by clonality tests [21]. Despite the co-expression of CD3 and CD20 observed in the present work, it is important to emphasize that a panel of three antibodies for B-cells was used, but we only anti-CD20 labelled the lymphoid cells. In fact, PAX 5 and CD79αcy are specific for particular stages of B-cell differentiation, but a good degree of overlapping is expected, which was not the case. The need to clarify the exact lineage of the lymphocytes involved in the process forced the clonality study, which revealed the presence of monoclonal rearrangement of the T-cell gamma receptor gene and the absence of amplification for B-cell receptors [12]. The diagnosis of EATL type 2 was finally confirmed.

In the present case, as in most of those already described [9,10,20], the definitive diagnosis of T-cell lymphoma required the identification of clonal rearrangement for gamma T-cell receptors (TCRγ) using the PARR technique. This only differs in one studied case of nodal lymphoma in which the same pattern of immunostaining was seen despite having clonal rearrangements of a similar amplitude of both T- and B-cell receptors (cross-lineage rearrangement), when applying the molecular method for the diagnosis; in this particular case, the molecular result led to the final classification of CD3+ CD20+ anaplastic lymphoma [11].

Aberrant expression of CD20 in T-cell lymphomas was also described in two cases of intestinal lymphoma associated with enteropathies in humans [22,23] and three cases in dogs [10]. Three hypotheses support possible causes for this co-expression in humans, but its origin and prognosis are still unclear and must be investigated in veterinary medicine. The first hypothesis supports the idea that the origin of this co-expression is a small population of T-cells that transcribe CD3 and CD20. These cells are mainly detected in peripheral blood and bone marrow and represent 3–5% of the total T-lymphocytes [24]. The second hypothesis is that CD20 expression on neoplastic T-cells is acquired during the malignant transformation of these cells. This is based on a study reporting that up to 60% of human transformed mycosis fungoides cases involve CD20 expression that was not initially present [25]. The third hypothesis then suggests a possible cross-reaction and instability of the anti-CD20 antibody, resulting in false-positive labelling of neoplastic T-cells. However, this hypothesis is considered unlikely, first because B-cells occupy their own specific territories in lymph nodes and are regularly used as positive controls, and also because this CD20 labelling of T-cells is seldom observed in the laboratory routine, being found only in neoplastic T-cells and not in others [26]. In the present case, this last hypothesis is out of the question because immunohistochemistry is performed in our laboratory on a weekly basis and the same antibody was applied to other samples at the same time without questionable results.

Type 1 enteropathy has been reported as predominant in intestinal T-cell lymphomas in dogs [6]. The co-expression of CD3 and CD20 in this neoplasm has already been described for the species, associated with type 1 enteropathy [10]. To the authors’ best knowledge, the present report is the first to refer to this co-expression in intestinal T-cell lymphoma associated with type 2 enteropathy, complicated by hepatic infiltration.

## 4. Conclusions

This case report reinforces the importance of combining different laboratory methods to accurately diagnose intestinal lymphomas and exposes a rarely reported presentation of co-expression of T- and B-cell markers in dog lymphoma, which should be considered in future investigations regarding its causes, clinical significance, and prognostic factor in the canine species.

## Data Availability

The data presented in this study are available on request from the corresponding author.
